# Mechanisms of Mitochondrial Dysfunction in Ageing and Neurodegenerative Diseases

**DOI:** 10.1002/alz70856_104338

**Published:** 2025-12-26

**Authors:** Shampa Ghosh, Krishna Kumar Singh, Amit Kumar Pandey, Suchita Jha, Jitendra Kumar Sinha

**Affiliations:** ^1^ GloNeuro Academy, Noida, Uttar Pradesh, India; ^2^ Symbiosis International (Deemed University), Pune, Maharastra, India; ^3^ Amity University Uttar Pradesh, Noida, UP, India

## Abstract

**Background:**

Mitochondrial dysfunction is an integral feature of both aging and neurodegenerative diseases, where it significantly contributes to disease progression. It is, therefore, of the utmost importance to understand the underlying mechanisms so that effective therapeutic approaches can be developed. This article delves into specific pathways where mitochondrial dysfunction occurs in aging and neurodegenerative conditions in the hope of discovering potential targets for intervention.

**Methods:**

A comprehensive literature review was done to synthesize current knowledge on mitochondrial dysfunction in ageing and neurodegeneration. It focused on the key cellular and molecular pathways that include changes in mitochondrial structure and function, disruptions in energy metabolism, and their impact on cellular homeostasis.

**Results:**

In short, the overall data point to complex interactions between the processes of aging, neurodegenerative disease and mitochondrial dysfunction. During aging, mitochondria become functionally less effective, characterized by decreased ATP levels, impaired oxidative phosphorylation and increased reactive oxygen species production. Different neurodegenerative diseases‐ Alzheimer's disease, Parkinson's disease, Huntington's disease‐had specific abnormalities in mitochondria, including deficient mitophagy, altered dynamics of mitochondria, and mutation in mitochondrial DNA. These cause neuronal cell death and accelerate progression of the diseases.

**Conclusion:**

This study elucidates the diverse mechanisms that connect mitochondrial dysfunction with both ageing and neurodegenerative diseases. Various pathways identified in this review are considered critical areas for therapeutic intervention aimed at preserving mitochondrial integrity and subsequently reducing detrimental impact of ageing and neurodegeneration on cellular function. These mechanisms offer more complex avenues for research into these critical pathways that should lead to novel treatments for age‐related and neurodegenerative conditions.

